# The role of diabetes in metastatic melanoma patients treated with nivolumab plus relatlimab

**DOI:** 10.1186/s12967-023-04607-4

**Published:** 2023-10-25

**Authors:** Domenico Mallardo, Rachel Woodford, Alexander M. Menzies, Lisa Zimmer, Andrew williamson, Egle Ramelyte, Florentia Dimitriou, Alexandre Wicky, Roslyn Wallace, Mario Mallardo, Alessio Cortellini, Alfredo Budillon, Victoria Atkinson, Shahneen Sandhu, Michielin Olivier, Reinhard Dummer, Paul Lorigan, Dirk Schadendorf, Georgina V. Long, Ester Simeone, Paolo A. Ascierto

**Affiliations:** 1 Melanoma, Cancer Immunotherapy, and Development Therapeutics Unit, Istituto Nazionale Tumori IRCCS “Fondazione G. Pascale”, Via Mariano Semmola, 53, 80131 Naples, Italy; 2 Melanoma Institute Australia, The University of Sydney, Royal North Shore and Mater Hospitals, Sydney, NSW Australia; 3 Department of Dermatology, University Hospital Essen, NCT-West, German Cancer Consortium, Partner Site Essen and University Alliance Ruhr, Research Center One Health, Essen, Germany; 4 Christie NHS Foundation Trust and Division of Cancer Services, University of Manchester, Manchester, UK; 5 Department of Dermatology, University of Zurich, Zurich, Switzerland; 6 Department of Oncology, Precision Oncology Center, Lausanne University Hospital, Rue du Bugnon 21, 1011 Lausanne, Switzerland; 7 Peter MacCallum Cancer Centre, Melbourne, Australia; 8 Greenslopes Private Hospital, University of Queensland QLD, Greenslopes, Australia; 9 Department of Surgery and Cancer, Imperial College London, Hammersmith Hospital, Du Cane Road, London, W120HS UK; 10 Operative Research Unit of Medical Oncology, Fondazione Policlinico Universitario Campus Biomedico, Rome, Italy; 11 Scientific Director, Istituto Nazionale Tumori IRCCS “Fondazione G. Pascale”, Naples, Italy

**Keywords:** Diabetes, Nivolumab + relatlimab, Melanoma, LDH

## Abstract

**Background:**

The combination of nivolumab + relatlimab is superior to nivolumab alone in the treatment of naive patients and has activity in PD-1 refractory melanoma. We had previously observed a reduced expression of *LAG3* in melanoma tissue from patients with type 2 diabetes.

**Method:**

*To* evaluate the impact of diabetes on oncological outcomes of patients with advanced melanoma treated with nivolumab plus the *LAG3* inhibitor relatlimab we performed a retrospective multicenter study.

**Results:**

Overall, 129 patients were included: 88 without diabetes before the treatment, 37 who were diagnosed with type 2 diabetes before the start of treatment, and 4 without diabetes before treatment who developed immune checkpoint inhibitor-induced diabetes (ICI-DM). PFS was 21.71 months (95% CI: 15.61–27.81) in patients without diabetes, 10.23 months (95% CI: 5.81–14.66) in patients with type 2 diabetes, and 50.85 months (95% CI: 23.04–78.65) in patients who developed ICI-DM. OS was 37.94 months (95% CI: 31.02–44.85) in patients without diabetes, 22.12 months (95% CI: 14.41–29.85) in those with type 2 diabetes and 57.64 months (95% CI: 42.29–72.99) in those who developed ICI-DM. Multivariate analysis showed that the presence of diabetes and LDH was correlated with OS and PFS. The mean OS was 64.63 months in subjects with low levels of glucose (< 137 mg/dl) and 36.27 months in those with high levels (hazard ratio 0.16, 95% CI: 0.04–0.58; p = 0.005). The patients whose glucose blood level increased after 3 months of treatment with nivolumab + relatinib compared to baseline (ratio of blood level at baseline/after 3 months > 1.5) had a worse prognosis than those whose glucose level had not increased. This result was observed also in subgroups treated either in first line or further lines. Patients who developed ICI-DM during the study period had better outcomes than the overall population and patients without diabetes.

**Conclusions:**

*LAG3* inhibition for treating metastatic or unresectable melanoma has a reduced efficacy in patients with type 2 diabetes, possibly due to a low expression of *LAG3* in tumor tissue. Higher level evidence should be obtained.

## Key message

*What is already known on this topic:* *LAG3* expression is reduced in melanoma tissue from patients with type 2 diabetes.

*What this study adds:* The low expression of *LAG3* is associated with reduced survival of patients with metastatic or unresectable melanoma and type 2 diabetes treated with *LAG3* inhibitor.

*How this study might affect research, practice or policy*: Patients with type 2 diabetes and metastatic melanoma are poor candidates for the treatment with the combination of nivolumab + relatlimab.

## Introduction

Immune checkpoint inhibitors (ICIs) have dramatically improved the outcomes for patients with advanced melanoma and are now a standard of care. Still, a substantial fraction of treated patients do not benefit long term from ICI treatment and loose tumor control. Novel combination regimens with new ICIs are being explored to enhance outcomes and reduce the risk of side-effects [1].

Lymphocyte-activation gene 3 (*LAG3*) and PD-1 are distinct inhibitory immune checkpoints often co-expressed on tumor-infiltrating lymphocytes and contributing to tumor-mediated T-cell exhaustion [2, 3]. In preclinical models, dual inhibition of LAG3 and PD-1 showed synergistic anti-tumor activity [3].

Relatlimab is a human IgG4 *LAG3*-blocking antibody that restores the effector function of exhausted T cells [4]. The combination of nivolumab + relatlimab showed anti-tumor activity, including durable objective responses, in patients with relapsed or anti-PD-1 refractory melanoma, in a phase I–II dose-escalation trial [5]. More recently, the phase II–III trial RELATIVITY-047 compared the fixed-dose combination of nivolumab + relatlimab with single agent nivolumab alone in patients with previously untreated metastatic or unresectable melanoma [6]. Progression-free survival (PFS) was superior in the combination arm compared to nivolumab (PFS; 10.1 months [95% CI: 6.4–15.7] vs 4.6 months [95% CI: 3.4–5.6]; HR 0.75 [95% CI: 0.62–0.92]; p = 0.006) [6]. Responses were observed regardless of *PD-1* and *LAG3* gene expression (1%), although enriched among patients with tumors expressing PD-L1 or *LAG3* [7].

Recently, our group conducted a gene profiling study of samples from peripheral blood of melanoma patients treated with ipilimumab, and found that patients with type 2 diabetes (T2D) had a lower expression of *LAG3* [8]. As diabetes is known to be an independent risk factor for several types of cancer, and diabetic subjects have higher cancer-related mortality compared to subjects without diabetes [9–11], our results suggested that the reduced expression of LAG3 could hinder the effect of anti-LAG3 treatments in diabetic patients with advanced melanoma.

This study aimed to evaluate the impact of diabetes on oncological outcomes in patients with advanced melanoma treated with nivolumab plus relatlimab.

## Patients and methods

### Study design

A pooled retrospective study with data obtained from eight centers in five countries was performed on 129 patients treated with nivolumab plus relatlimab. The study was performed in accordance with the revised version of the Declaration of Helsinki (52nd WMA General Assembly, Edinburgh, Scotland, October 2000).

Consecutive adult patients with metastatic melanoma at unresectable stage IIIb–IV and histologically confirmed, treated with fixed-dose combination of nivolumab plus relatlimab, in any line of treatment, aged over 18 years were enrolled. All patients provided their written informed consent.

The presence of diabetes was detected at hospitalization by the measure of glycosylated haemoglobin or of fasting blood glucose level (mean value of 3 measures, obtained once at baseline, and twice during the 8 weeks before treatment). Glucose level was also measured after 3 months of treatment with nivolumab plus relatlimab. A cut-off value for glucose level was assessed through Youden’ s J index, which maximizes sensitivity and specificity in a ROC curve, whose endpoint was death. Glucose level was classified as high when > 137 mg/dl. Change of glucose levels vs baseline was assessed after 3 months, and patients with a glucose level ratio > 1.5 were compared o those with ratio ≤ 1.5.

### Evaluation of outcomes

RECIST 1.1 criteria were used to evaluate the tumor response as complete response (CR), partial response (PR), stable disease (SD), or progressive disease (PD). The following parameters were recorded: response rate at first assessment, PFS (defined as the time from the administration of the first dose of checkpoint inhibitor to documented radiological progression, death or lost to follow-up, whichever occurred first), overall survival (OS; defined as the time from the administration of the first dose of checkpoint inhibitor to death or lost-to-follow-up, whichever occurred first), disease control rate (DCR; defined as the sum of CR, PR, and SD > 1 year), objective response rate (ORR; defined as the sum of CR and PR), Eastern Cooperative Oncology Group performance status (ECOG PS), American Joint Committee on Cancer (AJCC) distant metastases category (M), fasting blood glucose, and lactate dehydrogenase (LDH) level.

### Statistical analysis

Demographic and clinical data were tabulated using descriptive statistics, differences in characteristics of patients between the groups were tested by t-test or Wilcoxon test (according to their distribution) and Pearson chi-squared test for continuous and categorical variables, respectively. PFS was calculated from the start of treatment with a checkpoint inhibitor to the evidence of progressive disease or death, whichever occurs first; OS was calculated from the start of treatment with a checkpoint inhibitor to death or censored at the last follow-up. Survival times were analyzed using the Kaplan–Meier method, and the log-rank test assessed differences among curves. Hazard ratios (HRs) and their 95% CIs were estimated using a Cox regression model. Spearman’s rho analysis and χ^2^ log test were used to evaluate the association of variables.

## Results

### Patient’s characteristics

Overall, 129 patients were included in the study, of which 104 (70%) were males, 120 (93%) were Caucasian, and 3 (2%) were Asian (Table 1). *BRAF* mutation was present in 32 (25%) patients, while 77 (60%) carried wild-type *BRAF*, and *BRAF* status was not available for 20 (15%) patients. ICI treatment was in the first line in 40 (31%) subjects, in the second line in 33 (25%), in the third in 32 (25%), and beyond the third line in 24 (19%). The ECOG PS was 0–1 in 113 (88%) patients and ≥ 2 in 16 (12%).

**Table 1:** Patients’ demographic and clinical characteristics before treatment

	Type 2 diabetes at baseline n = 37, n (%)	No diabetes during observation n = 92, n (%)	Total population n = 129, n (%)	p-value
*Baseline patient characteristics*				
Mean age, years (range)	64 (17–85)	60 (26–94)	61 (17–94)	
Gender				0.9
Female	7 (19)	18 (20)	25 (30)	
Male	30 (81)	74 (80)	104(70)	
Race and ethnicity				0.0001
Caucasian	29 (78)	91 (99)	120 (93)	
Asian	2 (4)	1 (1)	3 (2)	
NA	6 (16)	0 (0)	6 (5)	
*BRAF* status				0.36
Wild-type	21 (57)	56 (61)	77 (60)	
Mutation	12 (32)	20 (22)	32 (25)	
NA	4 (11)	16 (17)	20 (15)	
*NRAS* status				0.41
Wild-type	15 (41)	40 (43)	55 (42)	
Mutation	5 (13)	6 (7)	11 (9)	
NA	17 (46)	48 (52)	63 (49)	
CNS metastases at baseline				0.19
Yes	4 (11)	16 (17)	20 (15)	
No	29 (78)	57 (62)	86 (69)	
NA	4 (11)	19 (21)	23 (16)	
M category				0.15
M1a	4 (11)	4 (4)	8 (6)	
M1b	7 (19)	10 (11)	17 (13)	
M1c	23 (62)	60 (65)	83 (66)	
M1d	3 (8)	18 (20)	21 (15)	
BMI, mean (SD)	29.7 (5.6)	27.11 (5.5)		0.01
T2D at baseline				–
Yes	37 (100)	0 (0)	37 (29)	
No	0 (0)	92 (100)	88 (62)	
Without diabetic episode during observation	–	–	88 (68)	
With ICI-DM development during treatment	–	–	4 (3)	
Antidiabetic treatments				–
Taking metformin	25 (68)	0 (0)	25 (19)	
Taking insulin	5 (14)	0 (0)	5 (4)	
Other hypoglycemic*	9 (24)	0 (0)	9 (7)	
NA	3 (5)	0 (0)	3 (2)	
Diabetic with glucose > 126 mg/dl**	19 (51)		19 (15)	
*Clinical parameters*				
ICI treatment				0.01
First-line treatment	15 (41)	25 (27)	40 (31)	
Second-line treatment	14 (38)	19 (21)	33 (25)	
Third-line treatment	3 (8)	29 (31)	32 (25)	
> Third-line treatment	5 (13)	19 (21)	24 (19)	
Previous treatments				
(Neo)adjuvant setting				0,35
Chemotherapy	2 (5)	2 (2)	4 (3)	
Targeted therapy	0 (0)	3 (3)	3 (2)	
Immunotherapy	4 (11)	7 (8)	11 (9)	
Metastatic setting:				
Chemotherapy	1 (3)	12 (13)	13 (10)	0.61
Targeted therapy	3 (8)	19 (21)	23 (18)	
Immunotherapy	18 (49)	52 (57)	70 (76)	
Systemic treatment post-nivolumab + relatlimab subministration	11 (30)	19 (21)	30 (12)	0.88
Immunotherapy	10 (27)	16 (17)	26 (21)	
Targeted therapy	1 (3)	2 (2)	3 (2)	
Chemotherapy	0 (0)	1 (1)	1 (1)	
ECOG PS				0.12
0–1	35 (95)	78 (85)	113 (88)	
≥ 2	2 (5)	14 (15)	16 (12)	
Concomitant radiotherapy	1 (3)	9 (10)	10 (8)	

^*^Glimepiride, sitagliptin, sodium-glucose cotransporter-2 (SGLT2), gliclazide, jardimet, linagliptin, sulfonylurea. **Fasting glucose levels were calculated on the average of 3 measurements, obtained once at baseline and twice during the 8 weeks before treatment

### Diabetes and oncologic outcomes

Among the 129 enrolled patients, 88 patients were not diabetic at the start of treatment and remained normoglycaemic throughout the observation period, 37 had T2D at the start of treatment, and four had no diabetes before treatment and developed ICI-induced diabetes (ICI-DM) during treatment. Patients without diabetes were more often Caucasian, had a lower mean BMI, and were more often on third and further lines of treatment than diabetic patients. Additionally, increased levels of LDH were found at baseline in 20/37 (54%) patients with diabetes and 21/62 (34%) subjects without diabetes (p < 0.001, Mann–Witney test).

**Fig. 1:**
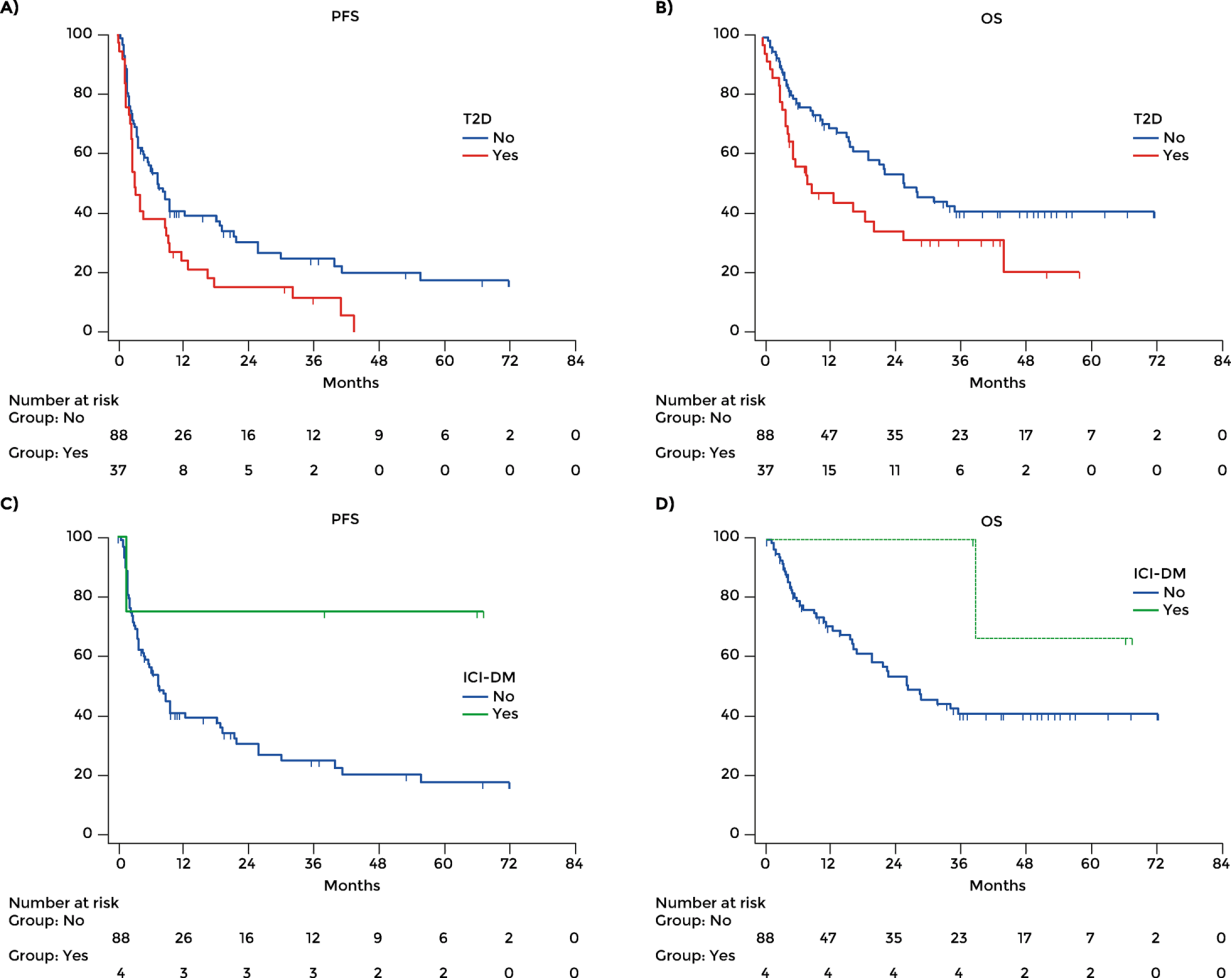
Overall study population: progression-free survival and overall survival according to the presence of type 2 diabetes (**A**, **B**) and ICI-DM, respectively (**C**, **D**)

PFS was 19.63 months (95% CI: 14.97–24.41) in the overall population, 21.71 months (95% CI: 15.61–27.81) in those without diabetes, 10.23 months (95% CI: 5.81–14.66) in patients with T2D, and 50.85 months (95% CI: 23.04–78.65) in patients who developed ICI-DM. The patients with T2D had a poorer mean PFS than patients without diabetes (HR 1.62, 95% CI: 1.01–2.60; p = 0.008) (Fig. 1A). All patients with T2D had progressed within 43 months of nivolumab + relatinib treatment.

**Fig. 2:**
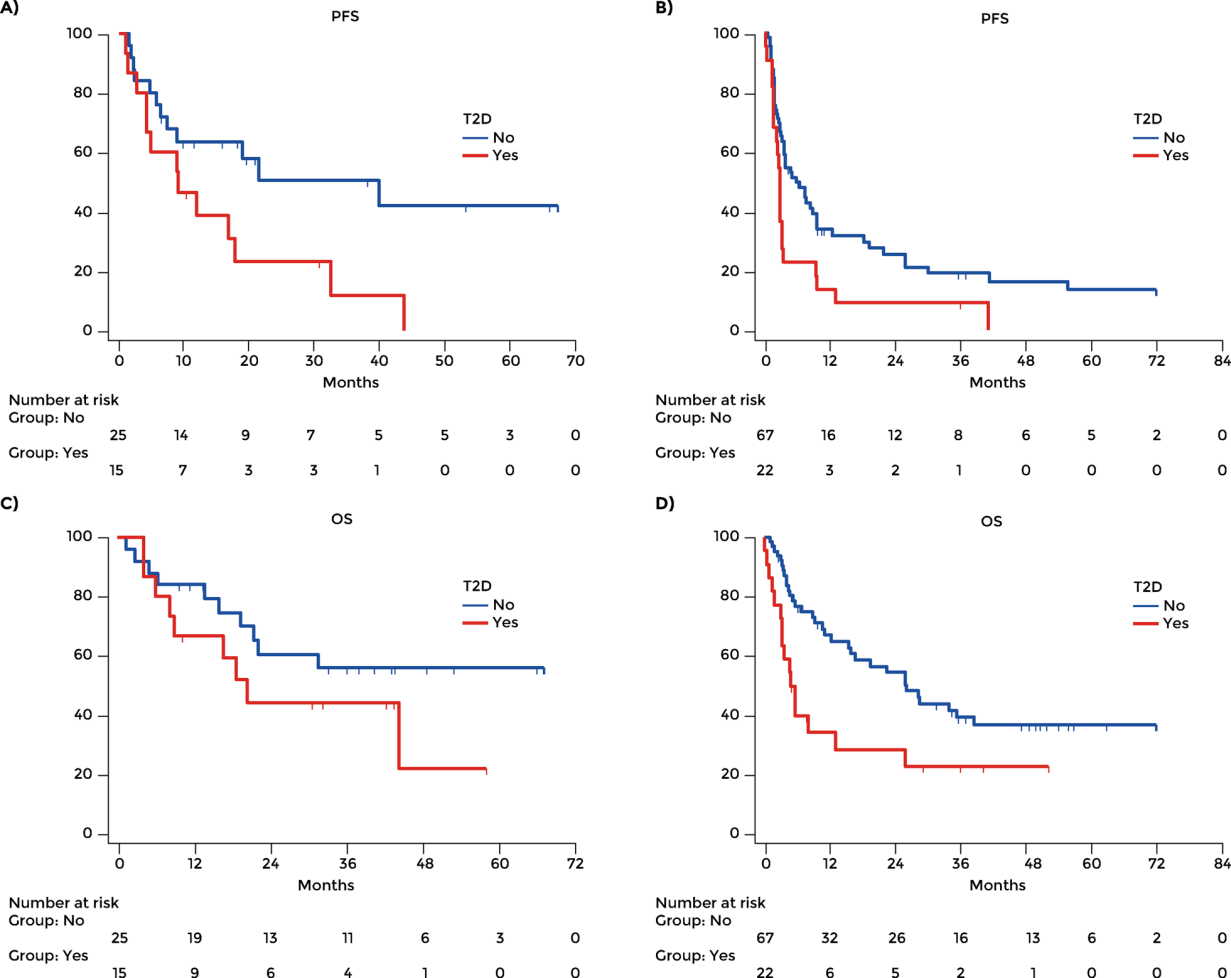
Progression free survival in patients treated in the first line (**A**) and in the second or further line (**B**). Overall survival in patients treated in the first line (**C**) and in the second or further line (**D**)

For patients treated in the first line, the mean PFS was 35.96 months in those without diabetes (95% CI: 23.59 – 48.33) and 14.86 months in those with T2D (95% CI: 7.32 – 22.50; p = 0.024).For patients treated in second or further line, the mean PFS was 18.28 months in those without diabetes (95% CI: 11.87 – 24.69) and 6.84 months in those with T2D (95% CI: 2.02 – 11.66; p = 0.012) (Fig. 2A, B).

The mean OS in the overall population was 35.30 months (95% CI: 29.65–40.95), 37.94 months (95% CI: 31.02–44.85) in those without diabetes (n = 88), 22.12 months (95% CI: 14.41–29.85) in those with T2D and 57.64 months (95% CI: 42.29–72.99) in those who developed ICI-DM during immunotherapy. The difference between patients with T2D and those without diabetes was significant (p = 0.029). The mean OS of patients with T2D was significantly shorter than in those without diabetes (HR 1.83, 95% CI: 1.05–3.17; p = 0.03) (Fig. 1B). For patients treated in the first line, the mean OS was 44.06 months in those without diabetes (95% CI: 33.03–55.08) and 29.00 months in those with T2D (95% CI: 17.64–40.35; p = 0.413). For patients treated in second or further line, the mean OS was 35.81 months in those without diabetes (95% CI: 27.86–43.76) and 16.40 months in those with T2D (95% CI: 7.47 – 25.33; p = 0.010) (Fig. 2C and D).

Although only four patients had ICI-DM, mean PFS was longer in patients with ICI-DM than in those without diabetes (HR 0.20, 95% CI: 0.08–0.49); also, these patients had better mean OS than the patients without diabetes (HR 0.30, 95% CI: 0.10–0.86) (Fig. 1C, D).

Multivariate analysis showed that the presence of diabetes and LDH was negatively correlated with PFS and OS in patients treated with nivolumab + relatlimab (Table 2).

**Table 2:** Univariate and multivariate analysis of oncologic risk factors correlating with progression-free survival and overall survival

Covariate	Univariate analysis			Multivariate analysis		
	p-value	HR	95% CI	p-value	HR	95% CI
*PFS*						
Gender	0.544	1.168	0.705–1.935	0.614	1.145	0.676–1.936
T2D	0.019	1.285	1.040–1.586	0.035	1.680	1.036–2.720
First-line treatment	0.033	0.607	0.384–0.960	0.050	0.614	0.377–1.000
ECOG PS > 2	0.660	1.131	0.651–1.966	0.345	1.343	0.727–2.476
LDH	0.002	1.906	1.253–2.900	0.021	1.706	1.080–2.692
*OS*						
Gender	0.780	1.085	0.610–1.930	0.613	1.165	0.643–2.108
T2D	0.032	1.310	1.022–1.679	0.050	1.727	1.000–2.981
First-line treatment	0.185	0.698	0.410–1.189	0.249	0.719	0.409 – 1.261
ECOG PS > 2	0.835	1.074	0.548–2.105	0.311	1.472	0.696–3.110
LDH	0.0001	2.683	1.617–4.451	0.0007	2.572	1.493–4.430

Univariate and multivariate analyses of comorbidities showed that T2D and obesity were negative prognostic factors for PFS and OS, while chronic pulmonary disease was a positive factor (Table 3).

**Table 3:** Univariate and multivariate analysis of comorbidities correlating with PFS and OS

Covariate	Univariate analysis			Multivariate analysis		
	p-value	HR	95% CI	p-value	HR	95% CI
*PFS*						
T2D	0.009	1.810	1.104–2.969	0.048	1.833	1.005–3.345
Peripheral vascular disease	0.664	0.818	0.446–1.499	0.887	0.961	0.555–1.662
Dementia	0.092	0.587	0.081–4.233	0.162	2.149	0.734–6.289
Renal disease	0.823	0.529	0.129–2.165	0.510	0.666	0.199–2.231
Obesity	0.060	1.913	1.121–3.263	0.451	1.230	0.717–2.109
Hypercholesterolemia	0.842	0.479	0.150–1.527	0.829	0.913	0.402–2.073
Hypertension	0.064	1.204	0.615–2.361	0.941	1.029	0.480–2.205
Thyroiditis	0.306	1.543	0.666–3.576	0.867	0.927	0.384–2.238
Chronic pulmonary disease	0.289	0.282	0.069–1.154	0.126	0.498	0.204–1.216
Other comorbidities	0.7791	1.112	0.654–1.890	0.507	1.180	0.722–1.929
*OS*						
T2D	0.018	1.740	1.142–2.651	0.009	2.377	1.240–4.557
Peripheral vascular disease	0.517	0.894	0.541–1.479	0.347	0.728	0.376–1.410
Dementia	0.597	2.389	0.866–6.590	0.386	0.408	0.053–3.093
Renal disease	0.376	0.892	0.327–2.430	0.263	0.393	0.076–2.019
Obesity	0.017	1.563	0.980–2.495	0.044	1.841	1.014–3.341
Hypercholesterolemia	0.213	0.929	0.450–1.917	0.334	0.527	0.143–1.934
Hypertension	0.586	1.645	0.971–2.786	0.212	0.551	0.216–1.406
Thyroiditis	0.311	1.463	0.705–3.033	0.417	1.517	0.554–4.153
Chronic pulmonary disease	0.078	0.638	0.278–1.463	0.046	0.228	0.053–0.977
Other comorbidities	0.692	1.066	0.681–1.668	0.252	1.388	0.791–2.435

### Blood glucose level and outcomes in patients with type 2 diabetes

**Fig. 3:**
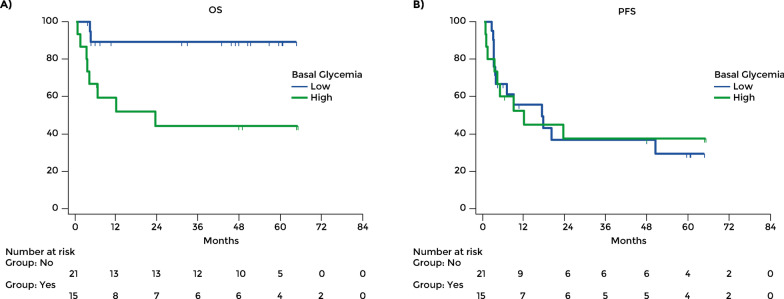
**A** Overall survival and **B** progression-free survival in patients with type 2 diabetes according to blood glucose level at baseline. Glucose level was classified as high when > 137 mg/dl

The role of glycemia level at baseline and its change during treatment were investigated. A cut off for the blood glucose level was determined by a ROC curve. In a subgroup of patients with T2D, outcomes of subjects with high glucose levels (≥ 137 mg/dl) were compared to those of subjects with low glucose levels (< 137 mg/dl). It was found that the subjects with low glucose levels (n = 21) had a better OS compared to those with high levels (n = 15) (Fig. 3A). The proportion of patients who died within the observation period was lower among those with low glycemia levels, 2 out of 21 (9.52%), than among those with high levels, 8/15 (53.33%). The mean OS was 64.63 months in subjects with low levels of glucose and 36.27 months in those with high levels (HR 0.16, 95% CI: 0.04–0.58; p = 0.005).

However, there was no difference in mean PFS (HR 1.03, 95% CI: 0.44–2.42; p = 0.94) (Fig. 3B).

**Fig. 4:**
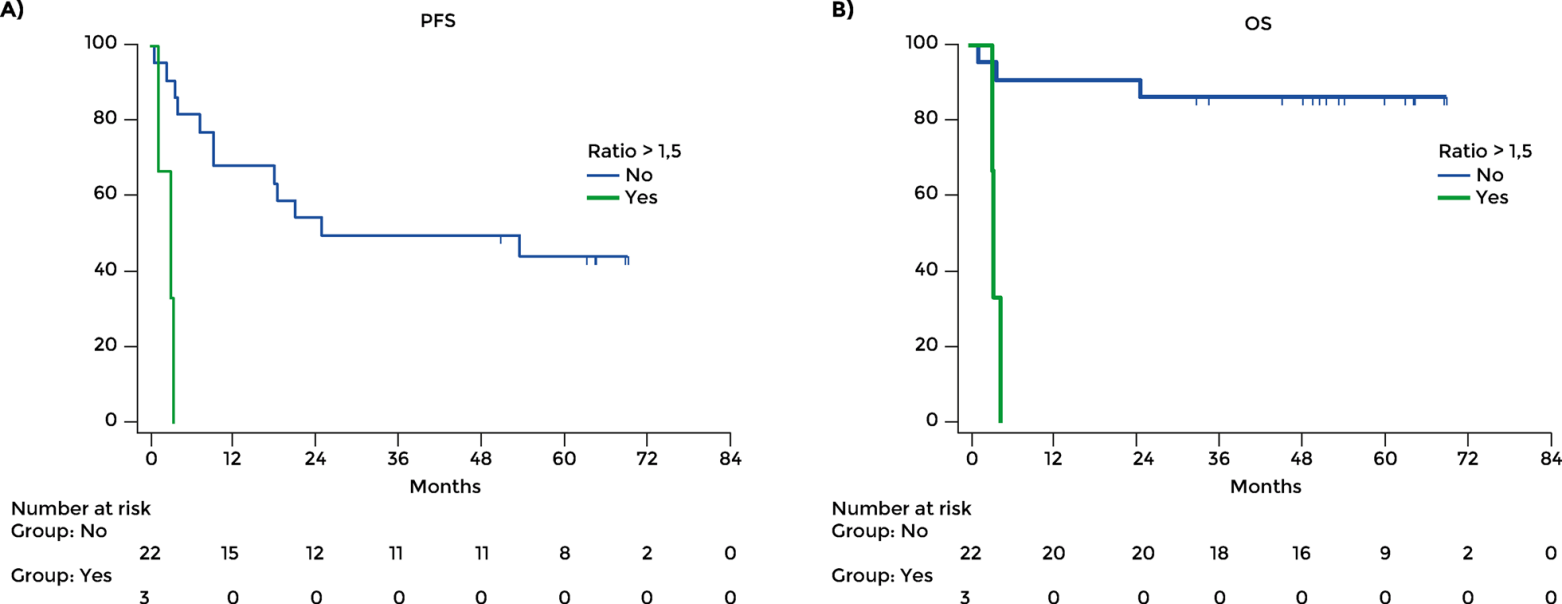
**A** Progression-free survival and **B** overall survival in patients with type 2 diabetes with increasing or stable blood glucose level during immunotherapy. Increasing level of glucose on treatment was defined as the ratio of blood level at baseline/after 3 months > 1.5

When glycaemic control in the first 3 months of treatment was examined in the T2D patients, those patients whose blood level increased after 3 months of treatment with nivolumab + relatinib compared to baseline (ratio of blood level at baseline/after 3 months > 1.5) had a worse prognosis compared to those whose glucose level had not increased (ratio of blood level at baseline/after 3 months ≤ 1.5) (Fig. 4). PFS was significantly different between these groups. The mean PFS was 40.72 months (95% CI: 28.03–53.40) in patients with stable glucose levels and 2.61 months in those with rising levels (HR 398.98, 95% CI: 19.55–8141.88; p = 0.0001). There were three deaths in the six patients with stable blood glucose, while all the 19 patients with rising levels died. The mean OS was 63.63 months (95% CI: 54.64–72.62) in patients with stable glucose levels and 3.97 (95% CI: 3.16–4.79; p < 0.0001) in those with rising levels.

**Fig. 5:**
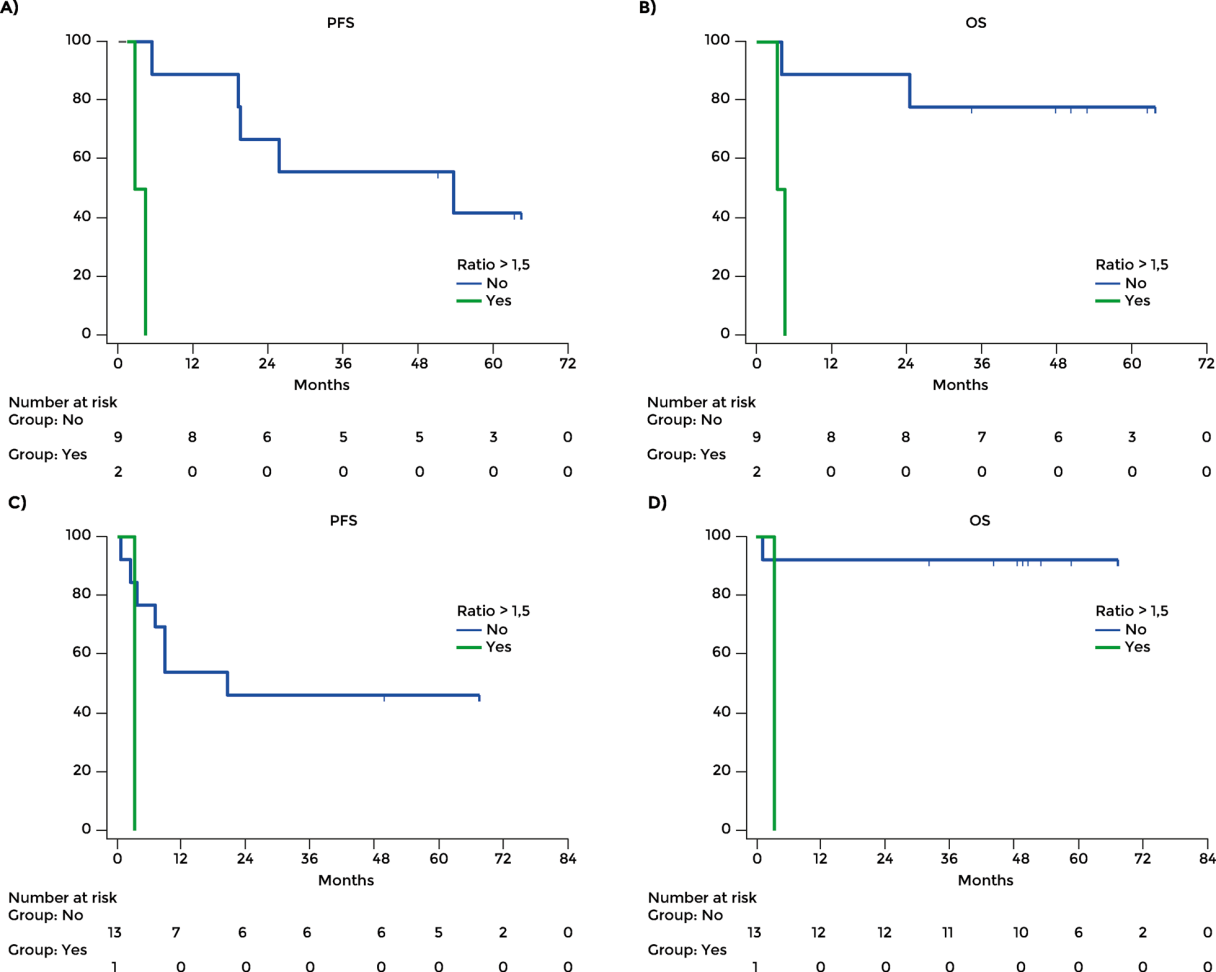
Change of glycemia level over 3 months of treatment. PFS (**A**) and OS (**B**) in patients treated in the first line. PFS (**C**) and OS (**D**) in patients treated in the second or further line

Similar results were obtained in patients treated in the first line. The mean PFS was 43.32 months with stable glycemia (95% CI: 27.35–59.29) and 2.18 months with rising levels (95% CI: 0.25–4.11; p = 0.0004) (Fig. 5A). OS was 55.63 months with stable levels (95% CI: 41.06–70.19) and 4.13 with rising levels (95% CI: 2.82–5.44; p = 0.005) (Fig. 5B). Among patients treated in the second or further line, the mean PFS was 37.49 with stable glycemia (95% CI: 19.91–55.07) and 3.46 with rising glycemia (95% CI: 3.46–3.46; p = 0.096) (Fig. 5C); the mean OS was 66.59 with stable glycemia (95% CI: 56.36–76.83) and 3.66 with rising glycemia (95% CI: 3.66–3.66; p = 0.02) (Fig. 5D).

## Discussion

This retrospective study of patients with advanced melanoma treated with the combination of nivolumab + relatlimab found that patients with T2D had worse oncologic outcomes compared to the whole population and subjects without diabetes. The analysis of subgroups of patients either treated in the first line or pre-treated confirmed the negative impact of T2D. Additionally, it was found that glucose level affected the negative prognostic relevance od T2D, and data suggest that control of glycemia throuout therapy with nivolumab + relatlimab may reduce the impact of diabetes on outcomes. Among patients with T2D, those with high baseline glucose levels at hospitalization (mean value out of 3 measurements ≥ 137 mg/dl) or with levels rising after 3 months of immunotherapy (ratio of blood level at hospitalization/after 3 months > 1.5) had worse prognosis than those with low baseline or stable levels. As expected, multivariate analysis showed that the presence of diabetes and LDH was negatively correlated with PFS and OS in patients treated with nivolumab + relatlimab.

These results may be examined in concomitance with our previous finding that patients with T2D have a reduced expression of *LAG3*, [8, 12] which could support a reduced efficacy of relatlimab.

T2D has an increasing prevalence in the population, and its impact on cancer risk and prognosis has been investigated by several authors [13–17]. Indeed, elevated blood glucose, insulin resistance, and obesity might play a crucial role in promoting carcinogenesis and impairing the effect of anti-tumor therapies [18, 19].

A recent retrospective cohort study on 382 patients with head and neck melanoma found that T2D is a relevant comorbidity and is associated with reduced 5-year recurrence-free survival and survival (p = 0.016), while therapy with metformin had a favorable effect reducing the risk of 5-year recurrence (p = 0.03) [20]. Straker et al. reported similar observations on cutaneous melanoma [21]. They found that patients with T2D had an increased risk of high thickness, satellitosis, and 5-year recurrence.

These data seem in agreement with our findings, as the improved prognosis of subjects treated with metformin could stand with the better outcomes found by us in patients with controlled diabetes vs those with higher levels of glucose. It must be acknowledged that current data cannot state whether T2D has a direct negative prognostic role or should be considered as a marker of poor performance status or sevre comorbidities. Thus, the clinical effect of glucose level control on tumor outcomes should be further investigated.

We observed four patients who developed ICI-DM following immunotherapy with nivolumab + relatlimab; this frequency was higher than reported in the literature, but this may not be significant, due to the reduced numerosity of this group, representing a limitation of our study [22]. Patients who developed ICI-DM during the observation period had better outcomes than the overall population and patients without diabetes. We can speculate that ICI-DM may be induced by the effect of LAG3 inhibition, which promotes CD4 and CD8 cell infiltration in tumors and other sites, including the pancreas. An in vivo experimental study demonstrated that 100% of *LAG3* knockout mice develop diabetes with a peak insulin level, suggesting rapid destruction of pancreatic β cells. These mice exhibited accelerated, invasive insulitis, with increased CD4 + and CD8 + T-cell islet infiltration [23]. Considering this evidence, the development of ICI-DM during LAG3 inhibition is an adverse event related to immunotherapy that might be considered a result of the beneficial immune activation due to the checkpoint inhibitor. Indeed, immune-related adverse events of immunotherapy for melanoma are frequent findings and are associated with longer OS in cancer patients receiving ICIs [24].

Although ICI-DM was reported in only 0.1% of the patients in clinical trials, its development may suggest a favorable response and be a guide for therapeutic decisions [22]. On the contrary, T2D has a high and rising prevalence in the general population and may be a frequent comorbidity in patients with advanced melanoma. Thus, it should be taken into account as warranting a reduced response to nivolumab plus relatlimab [25, 26].

Contrary to previous reports [27, 28], we found that a high body weight was associated with poor OS in melanoma patients receiving ICI. We speculate that obesity in our patients was associated with T2D, which exceeded the positive effect of the immunogenic phenotype associated with high BMI. Conversely, comorbidity with chronic pulmonary disease was a positive factor for OS; it is possible to speculate that chronic inflammation facilitates an anti-tumor immunologic response.

In conclusion, LAG3 inhibition for treating metastatic or unresectable melanoma has a reduced efficacy in subjects with T2D, possibly due to a low expression of *LAG3*. Although current evidence cannot prove the causative role of glycemia on reduced efficacy of LAG3 inhibition, data suggest that glycemia should be monitored and maintained within control levels in these patients. The development of ICI-DM during *LAG3* inhibition might indicate the high efficacy of immunotherapy through an important immune activation.

## Data Availability

Data and material have been deposited and are publicly available at https://doi.org/10.5281/zenodo.8031847.

## Abbreviations

AJCC: American Joint Committee on Cancer
CR: Complete response
DCR: Disease control rate
ECOG PS: Eastern Cooperative Oncology Group performance status
HR: Hazard ratio
ICI: Immune checkpoint inhibitor
ICI-DM: Immune checkpoint inhibitor-induced diabetes mellitus
*LAG3*: Lymphocyte-activation gene 3
LDH: Lactate dehydrogenase
ORR: Objective response rate
OS: Overall survival
PD: Progressive disease
PFS: Progression-free survival
PR: Partial response
SD: Stable disease
T2D: Type 2 diabetes
